# Long-Term Effects, Pathophysiological Mechanisms, and Risk Factors of Chemotherapy-Induced Peripheral Neuropathies: A Comprehensive Literature Review

**DOI:** 10.3389/fphar.2017.00086

**Published:** 2017-02-24

**Authors:** Nicolas Kerckhove, Aurore Collin, Sakahlé Condé, Carine Chaleteix, Denis Pezet, David Balayssac

**Affiliations:** ^1^INSERM U1107, NEURO-DOL, CHU Clermont-Ferrand, Délégation à la Recherche Clinique et à l’Innovation, Université Clermont AuvergneClermont-Ferrand, France; ^2^INSERM U1107, NEURO-DOL, Université Clermont AuvergneClermont-Ferrand, France; ^3^INSERM U1107, NEURO-DOL, CHU Clermont-Ferrand, Neurologie, Université Clermont AuvergneClermont-Ferrand, France; ^4^CHU Clermont-Ferrand, Hématologie Clinique AdulteClermont-Ferrand, France; ^5^INSERM U1071, CHU Clermont-Ferrand, Chirurgie et Oncologie Digestive, Université Clermont AuvergneClermont-Ferrand, France

**Keywords:** chemotherapy-induced peripheral neuropathy, long-term effects, pathophysiological mechanisms, risk factors, anticancer drugs

## Abstract

Neurotoxic anticancer drugs, such as platinum-based anticancer drugs, taxanes, vinca alkaloids, and proteasome/angiogenesis inhibitors are responsible for chemotherapy-induced peripheral neuropathy (CIPN). The health consequences of CIPN remain worrying as it is associated with several comorbidities and affects a specific population of patients already impacted by cancer, a strong driver for declines in older adults. The purpose of this review is to present a comprehensive overview of the long-term effects of CIPN in cancer patients and survivors. Pathophysiological mechanisms and risk factors are also presented. Neurotoxic mechanisms leading to CIPNs are not yet fully understood but involve neuronopathy and/or axonopathy, mainly associated with DNA damage, oxidative stress, mitochondria toxicity, and ion channel remodeling in the neurons of the peripheral nervous system. Classical symptoms of CIPNs are peripheral neuropathy with a “stocking and glove” distribution characterized by sensory loss, paresthesia, dysesthesia and numbness, sometimes associated with neuropathic pain in the most serious cases. Several risk factors can promote CIPN as a function of the anticancer drug considered, such as cumulative dose, treatment duration, history of neuropathy, combination of therapies and genetic polymorphisms. CIPNs are frequent in cancer patients with an overall incidence of approximately 38% (possibly up to 90% of patients treated with oxaliplatin). Finally, the long-term reversibility of these CIPNs remain questionable, notably in the case of platinum-based anticancer drugs and taxanes, for which CIPN may last several years after the end of anticancer chemotherapies. These long-term effects are associated with comorbidities such as depression, insomnia, falls and decreases of health-related quality of life in cancer patients and survivors. However, it is noteworthy that these long-term effects remain poorly studied, and only limited data are available such as in the case of bortezomib and thalidomide-induced peripheral neuropathy.

## Introduction

Platinum-based anticancer drugs (i.e., cisplatin, oxaliplatin), proteasome/angiogenesis inhibitors (bortezomib/thalidomide), vinca alkaloids (i.e., vincristine, vinorelbine) and taxanes (i.e., paclitaxel, docetaxel) are the most common anticancer drugs used as first-line chemotherapy for several cancers, including colorectal, gastric, breast and lung cancers, and multiple myeloma. Despite their different action mechanisms, all these anticancer drugs share a common adverse and disabling effect for patients, namely CIPN (Balayssac et al., 2011). CIPN has a considerable impact on cancer treatments and their related symptoms severely affect patients’ daily activities and quality of life. Thus CIPN is often the main adverse effect leading to the reduction or discontinuation of chemotherapy. Moreover, these symptoms may continue to develop and progress for several months post-therapy (so called “coasting effect”) and may persist over periods lasting from several months to years after ceasing chemotherapy (Argyriou et al., 2012). Classic clinical symptoms of CIPN involve the PNS and lead to peripheral neuropathy with a “stocking and glove” distribution characterized by sensory loss, paresthesia, dysesthesia, numbness, and tingling sometimes associated with neuropathic pain in the most serious cases (Balayssac et al., 2011; Jaggi and Singh, 2012). Today, the overall incidence of CIPN is estimated at approximately 38% (possibly up to 90% of patients treated with oxaliplatin) (Cavaletti and Zanna, 2002; Balayssac et al., 2011). Unfortunately, there is no preventive or curative treatment for CIPN at present (Hershman et al., 2014). Finally, many of these neurotoxic anticancer drugs are used in both adjuvant and palliative settings. These neurotoxic drugs raise issues in the case of adjuvant settings, because CIPN may last for a long time after the end of chemotherapy, with very negative impacts on cancer survivors. These effects are well-described for the use of adjuvant oxaliplatin-based chemotherapy for colorectal cancer, for which patients may have long life expectancy (Tofthagen, 2010; Stefansson and Nygren, 2016). The health impacts of CIPN remain worrying, because CIPN is associated with comorbidities such as psychological distress, fall risk and sleep disorders (Hong et al., 2014). Moreover, CIPN affects a specific population of patients already impacted by cancer, which is a strong driver for declines in physical functioning and increased risk of depression in older adults (Leach et al., 2016). Finally, CIPN represents a heavy economic burden. For example, in the USA, on average CIPNs increase healthcare costs by $17,344 per year per patient (Pike et al., 2012).

The purpose of this review is to present a comprehensive overview of the long-term effects of CIPN in cancer patients and survivors. Pathophysiological mechanisms and risk factors are also presented.

## Platinum-Based Anticancer Drugs

Platinum-based anticancer drugs are composed of cisplatin, carboplatin and oxaliplatin, which are the main authorized anticancer drugs. Platinum-based anticancer drugs act through DNA platination which interferes with cell viability and division (Dilruba and Kalayda, 2016). Cisplatin and carboplatin are indicated in several solid cancers such as of the lung, ovary, testes and uterus, whereas oxaliplatin is indicated for tumors of the digestive tract, mainly in advanced colorectal cancers (Brenner et al., 2014) but also of the esophagus, stomach, liver, and pancreas (Javle and Hsueh, 2010). Cisplatin and oxaliplatin are probably more neurotoxic than carboplatin (McWhinney et al., 2009).

### Pathophysiological Mechanisms of CIPN Associated to Platinum-Based Anticancer Drugs

Platinum-based anticancer drugs reach the neurons of the PNS and induce several types of toxic effects, among them nuclear and mitochondrial DNA damage, oxidative stress and ion channel disturbances. Platinum-based anticancer drugs are alkylating drugs capable of causing nuclear damage. This damage, such as to DNA cross-links, have been directly correlated to electrophysiological abnormalities in peripheral nerves (Dzagnidze et al., 2007). Such DNA damage may also affect mitochondrial DNA. Cisplatin was observed to inhibit the replication and transcription of mitochondrial DNA, and was responsible for mitochondrial degradation (Podratz et al., 2011). Lastly, cisplatin was seen to induce pro-apoptotic changes in the sciatic nerves of cisplatin-treated mice (Sharawy et al., 2015).

Platinum derivatives are also responsible for oxidative stress in PNS neurons. Oxaliplatin treatment has been observed to increase protein carbonylation and lipid peroxidation in both the sciatic nerves and the spinal cord. This oxidative stress was reduced by antioxidants such as silibinin and α-tocopherol (Di Cesare Mannelli et al., 2012). MnL4, a superoxide dismutase mimetic compound, decreased superoxide anion production, lipid peroxidation and intracellular calcium signals induced by oxaliplatin *in vitro*. MnL4 decreased mechanical hyperalgesia, and mechanical and cold allodynia induced by oxaliplatin in rats (Di Cesare Mannelli et al., 2016). Oxidative stress has also been observed in sciatic nerves in cisplatin-treated mice, with an increase of malondialdehyde and a decrease of superoxide dismutase and glutathione (Sharawy et al., 2015).

**FIGURE 1:**
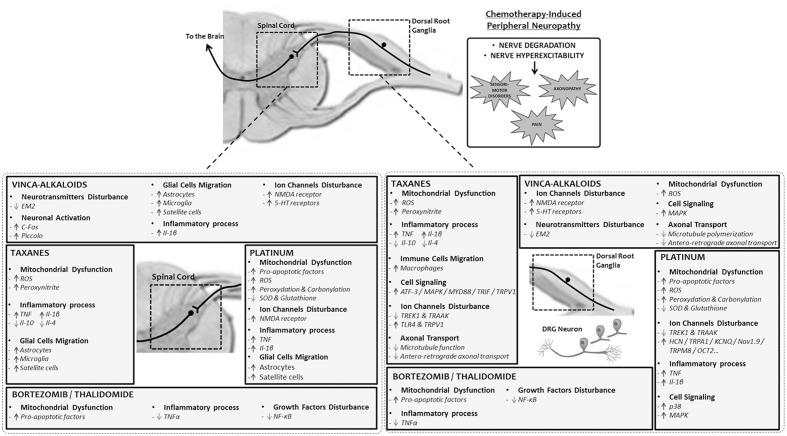
**Mechanisms of chemotherapy-induced peripheral neuropathy.** Pathophysiological alterations triggered by platinum, taxanes, vinca-alkaloids, and bortezomib/thalidomide in the peripheral nervous system, dorsal root ganglia and spinal cord. 5-HT, 5-hydroxytryptamine; ATF-3, cyclic AMP-dependent transcription factor 3; EM2, endomorphin-2; HCN, potassium/sodium hyperpolarization-activated cyclic nucleotide-gated channel; Il-1β, interleukin-1β; Il-10, interleukin-10; Il-4, interleukin-4; KCNQ, potassium channel, subfamily Q; MAPK, mitogen-activated protein kinase; MYD88, myeloid differentiation primary response gene 88; Nav1.9, voltage-gated sodium channel member 1.9; NMDA, *N*-methyl-D-aspartate; NF-κB, nuclear factor-kappa B; OCT2, solute carrier family 22 member 2 (organic cation transporter); ROS, reactive oxygen species; SOD, superoxide dismutase; TLR4, toll-like receptor 4; TNF, tumor necrosis factor; TRAAK, TWIK-related arachidonic acid activated K^+^ channel; TREK1, TWIK1-related K^+^ channel 1; TRIF, TIR-domain-containing adapter-inducing interferon-β; TRPA1, transient receptor potential cation channel, subfamily A, member 1; TRPM8, transient receptor potential cation channel subfamily M member 8; TRPV1, transient receptor potential vanilloid 1.

Ion channels are also a toxic target of platinum-based anticancer drugs. These interactions with ion channels have mainly been studied for oxaliplatin. A single administration of oxaliplatin to mice induced neuronal hyperexcitability, decreasing the expression of potassium channels, TREK1, and TRAAK (TWIK refers to a tandem of P domains in a weak inward-rectifier K^+^ channel), and increasing HCN channels in DRG neurons (Descoeur et al., 2011). More specifically, several ion channels act as thermal sensors which are involved with oxaliplatin-induced thermal hyperesthesia. Oxaliplatin induced cold hyperalgesia in rats through the activation of transient receptor potential ankyrin 1 and p38 MAPK (p38 mitogen-activated protein kinase) in DRG neurons (Yamamoto et al., 2015). In trigeminal ganglion neurons, the inhibition of KCNQ (potassium voltage-gated channel subfamily KQT) channels, voltage-gated K^+^ channels mediating M-currents, suppressed oxaliplatin-induced orofacial cold hyperalgesia in rats (Abd-Elsayed et al., 2015). Disruption of the voltage-gated sodium channel NaV1.9 in mice suppressed oxaliplatin-induced cold allodynia and hyperalgesia (Lolignier et al., 2015). Oxaliplatin induced an increase of the cool sensor TRPM8 expression in DRG neurons in rats. Oxaliplatin-induced cold allodynia was suppressed by TRPM8 inhibition (Gauchan et al., 2009). Likewise, cisplatin seems to be able to interfere with ion channels in the DRG neurons of rats, by decreasing voltage-gated potassium and calcium channel currents (Tomaszewski and Büsselberg, 2007) (**Figure 1**).

### Symptoms and Long-Term Effects of Oxaliplatin-Induced Peripheral Neuropathy

Oxaliplatin has strong neurotoxicity which is qualitatively and quantitatively different from other neurotoxic anticancer drugs (Balayssac et al., 2011). Oxaliplatin is responsible for acute neuropathic disturbances (paresthesia, dysesthesia of the hands, feet and perioral area induced by cold stimuli) occurring in the hours or days after chemotherapy infusion (Balayssac et al., 2011). At the beginning of chemotherapy cycles, this acute neuropathy usually resolved by itself within a week and disappeared for the next chemotherapy cycle. But the repetition of chemotherapy cycles induced chronic and invalidating CIPN for several patients (Balayssac et al., 2011). This chronic CIPN is associated with paresthesia, numbness, sensory ataxia and can lead to functional deficits (Zedan et al., 2014). Cold hyperesthesia is characteristic of acute oxaliplatin-induced peripheral neuropathy and may augur severe chronic neuropathy (Attal et al., 2009). This CIPN may be aggravated by cold external temperatures, such as in Nordic countries (Altaf et al., 2014; Stefansson and Nygren, 2016) (**Table 1**).

**Table 1:** Main symptoms associated with chemotherapy-induced peripheral neuropathy.

**Platinum-based anticancer drugs**
Oxaliplatin	Acute CIPN (>90% of patients): paresthesia, dysesthesia of the hands, feet and perioral area induced by cold stimuli
	Chronic CIPN (30–50% of patients): paresthesia, numbness, sensory ataxia, functional deficits, and pain
	No vegetative disturbances
	Coasting effect
	Maximum duration in the literature: 8 years
Cisplatin	Sensory neuropathy similar to oxaliplatin-induced chronic neuropathy (50% of patients)
	Maximum duration in the literature: 25 years (adult survivors of childhood extracranial solid tumors)
**Taxanes**
Paclitaxel	80–97% of patients
Docetaxel	Acute and chronic sensory neuropathy associated with paresthesia, numbness, tingling and burning, and mechanical and cold allodynia
	Rare motor symptoms with mild distal weakness and myalgia
	Rare vegetative disturbances
	Coasting effect
	Maximum duration in the literature: 4.75 years
**Vinca alkaloids**
Vinblastine	35–45% of patients
Vinorelbine	Sensory neuropathy in the hands and feet, leading to functional disability with fine motor tasks and walking, including numbness and tingling
Vindesine	Motor neuropathy with cramps and distal muscle weakness
Vincristine	Vegetative neuropathy associated with postural hypotension, bladder and bowel disturbance
	Coasting effect
	Maximum duration in the literature: 7 years (cancer survivors of childhood hematological malignancies)
**Proteasome inhibitor**
Bortezomib	31–64% of patients
	Sensory neuropathy associated with burning dysesthesia, coldness, numbness, hyperesthesia, and/or tingling in a distal stocking-and-glove distribution over the hands and feet
	Pain
	Vegetative disturbances
	Maximum duration in the literature: 2 years (little data in the literature)
**Immunomodulatory**
Thalidomide	10–55% of patients
	Sensory peripheral neuropathy associated with tingling or painful paresthesia, and numbness in the lower limbs
	Mild motor impairments
	Vegetative disturbances including gastrointestinal (constipation, anorexia, and nausea) and cardiovascular (hypotension and bradycardia) manifestations
	Maximum duration in the literature: no clear information (little data in the literature)


Oxaliplatin is probably the most neurotoxic anticancer drug since more than 90% of patients developed acute neuropathy and 30–50% of patients developed chronic neuropathy (Tofthagen, 2010; Beijers A.J. et al., 2014). Grade severity and symptom duration vary between studies (Beijers A.J. et al., 2014). Although symptoms decrease with time, long-term clinical studies seem to demonstrate the persistence of neuropathy after 24 months (Beijers A.J. et al., 2014): 25 months, 37.5% of grade 1, 29.2% of grade 2 and 0.7% of grade 3 (Park et al., 2011); 48 months, 11.9% of grade 1, 2.8% of grade 2 and 0.7% of grade 3 (André et al., 2009); 8 years, 30.4% of grade 2+ (Yothers et al., 2011). Consequently, the reversibility of this CIPN remains equivocal (Park et al., 2011). Moreover, some authors have suggested that oxaliplatin-induced peripheral neuropathy could be more frequent and more severe in the long-term than expected, lasting more than 12 months (Vatandoust et al., 2014); however, these patients represent a third of the population of cancer survivors (Ganz, 2003).

In colorectal cancer survivors, CIPN has a strong negative impact on HRQOL, associated with depression and sleep disorders (Mols et al., 2013; Tofthagen et al., 2013). More worrying, some cancer survivors may feel “poisoned” by chemotherapy, for more details see the patient’s comments in Tofthagen (2010).

### Symptoms and Long-Term Effects of Cisplatin-Induced Peripheral Neuropathy

In clinical practice, cisplatin induced neuropathy is similar to chronic oxaliplatin-induced peripheral neuropathy; it remains a sensory axonal neuropathy with abnormal nerve conduction and no remarkable vegetative disturbances (Earl et al., 1998; Balayssac et al., 2011).

The prevalence and long-term effects of this CIPN have been assessed in several studies and in different types of cancer. In the case of ovarian cancer, it has been reported that 50% of patients treated with cisplatin-based chemotherapy complained of peripheral sensory neuropathy after a median of 5.7 years (minimum: 5 months, maximum: 17 years) (Engelen et al., 2009). In the case of adolescents and young adults treated for bone and soft tissue sarcomas, approximately half of the patients presented a CIPN after an 8-month median (minimum: 1 month, maximum: 54 months) (Earl et al., 1998). In the case of testicular cancer, about 38% of patients had non-symptomatic neuropathy at a median of 15 years after cisplatin-based chemotherapy (minimum: 13, maximum: 17 years), 28% had symptomatic neuropathy and 6% had a disabling polyneuropathy (Strumberg et al., 2002). In another study, 29% of patients had paresthesia in their hands and feet 10.7 years after the end of cisplatin-based chemotherapy (minimum: 4, maximum: 21 years) (Brydoy et al., 2009). Glendenning et al. (2010) found that after a median of 11 years (minimum: 3 years, maximum: 19 years), 21.7% of testicular cancer survivors presented a peripheral neuropathy. In adult survivors of childhood extracranial solid tumors, sensory and motor neuropathies were detected in 20 and 17.5% of patients respectively, after a median time since cancer diagnosis of 25.2 years (minimum: 10.7 years, maximum: 48.2 years). In these patients who had received several lines of different chemotherapies, motor impairment was related to vinca alkaloid whereas sensory impairment was related to platinum-based drug exposure. Sensory neuropathy was associated with a decrease of endurance and mobility (Ness et al., 2013).

### CIPN Risk Factors Associated to Platinum-Based Anticancer Drugs

Several risk factors have been identified for these platinum-based anticancer drug-associated CIPN. Cumulative dose is the main risk factor for platinum-based drugs, >850 mg/m^2^ for oxaliplatin (Beijers A.J. et al., 2014) and >200–300 mg/m^2^ for cisplatin (Earl et al., 1998; Glendenning et al., 2010). Pre-treatment anemia, hypoalbuminemia and hypomagnesaemia and alcohol consumption have been identified as risk factors for oxaliplatin-induced peripheral neuropathy (Vincenzi et al., 2013). Gender, hypocalcaemia, diabetes, and chronic renal failure were not associated with CIPN (Vincenzi et al., 2013). Radiotherapy may increase the neurological symptoms for cisplatin-induced peripheral neuropathy (Brydoy et al., 2009).

Several studies have also assessed genetic predisposition to or protection against CIPN, mainly through polymorphisms affecting the pharmacokinetics of platinum-based anticancer drugs. Thus cisplatin-induced peripheral neuropathy was less frequent in patients with GST M1 (deletion) or GSTM3 intron 6 AGG/AGG genotypes (Khrunin et al., 2010). Conversely, SNP affecting the GSTP1 (IIe105Val) and GSTM1 (deletion) genotypes were significantly associated with a higher incidence of oxaliplatin-associated CIPN (grade > 2) (Kumamoto et al., 2013). SNPs affecting cyclin H and the BCRP were significantly associated with a higher risk of severe oxaliplatin-induced peripheral neuropathy (Custodio et al., 2014). Interestingly, NaV polymorphisms could also be associated with both the severity of acute oxaliplatin-induced neuropathy and the occurrence of chronic neuropathy (Argyriou et al., 2013). However, although several studies have been performed on this topic, the impact of SNP on platinum-based anticancer drugs remains equivocal, since a meta-analysis was unable to find any association between GSTP1 IIe105Val and oxaliplatin-induced peripheral neuropathy (Peng et al., 2013). Furthermore, Terrazzino et al. (2015) did not find any association between eight selected SNPs and oxaliplatin-induced peripheral neuropathy (**Table 2**).

**Table 2:** Probable risk factors by type of anticancer drug.

Probable risk factors	Platinum-based anticancer drugs	Taxanes	Vinca alkaloids	Bortezomib thalidomide
Chemotherapy regimen	Cumulative dose	Dose intensity	Cumulative dose	>1 mg/m^2^
	>850 mg/m^2^ (oxaliplatin)		>2 mg/m^2^	Induction therapy
	>200–300 mg/m^2^ (cisplatin)			
Medical History	Pre-treatment anemia	Pre-existing neuropathy	CMT1A	Pre-existing neuropathy
	Hypoalbuminemia			
	Hypomagnesaemia			
	Radiotherapy			
	Pre-existing neuropathy			
Genetic factors (SNP)	GSTP1 (IIe105Val)	FGD4	CYP3A5 GLI1	–
	GSTM1 (deletion)	EPHA5	CEP72	
	cyclin H	FZD3		
	BCRP	CYP2C8		
	Na_V_ channels	CYP3A5^∗^3		
		CYP3A4^∗^22		
		ABCB1		
Demographic variables	Age	Age	Age (children)	–


*Na_v_, voltage-gated sodium channel; GSTP1, glutathione *S*-transferase pi 1; GSTM1, glutathione *S*-transferase mu 1; BCRP, ATP-binding cassette sub-family G member 2 (ABCG2); FGD4, FYVE, RhoGEF and PH domain containing 4; EPHA5, ephrin type-A receptor 5; FZD3, Frizzled-3; CYP2C8, cytochrome P450 2C8; CYP3A5, cytochrome P450 3A5; CYP3A4, cytochrome P450 3A4; ABCB1, ATP-binding cassette sub-family B member 1; CMT1A, Charcot-Marie-Tooth type 1A; CEP72, centrosomal protein 72.*

## Taxanes

Taxane diterpenoids were isolated from the bark of the Pacific yew tree (*Taxus brevifolia* and *Taxus baccata* for paclitaxel and docetaxel, respectively) (Saloustros et al., 2008; Wani and Horwitz, 2014). Taxanes have been approved by the US Food and Drug Administration (FDA) since the mid-1990s for the treatment of several cancers: breast, ovarian, non-small cell lung, prostate, gastric, and head/neck (Qin et al., 2012).

Taxanes are microtubule-stabilizing drugs, thus preventing their depolymerization (Amos and Löwe, 1999; Zhang et al., 2014). This stabilization promotes the formation of abnormal bundles of microtubules in the cytoplasm, leading to mitotic spindle disruption. Thus cells arrest their cell cycle in the G0/G1 and G2/M phases, leading to apoptosis in dividing cells (mainly tumor cells) (Hornick et al., 2008).

### Pathophysiological Mechanisms of CIPN Associated to Taxanes

The pathogenesis of peripheral neuropathy induced by taxanes has been investigated in numerous studies (Höke and Ray, 2014). Nevertheless, the primary site of pathogenesis of taxane-associated CIPN has not yet been elucidated (Gornstein and Schwarz, 2014). The increase of oxidative stress may contribute to the potent neurotoxicity of taxanes through damage to neuronal and non-neuronal cells in the PNS, macrophage activation in the DRG and peripheral nerves, and microglial activation in the spinal cord (Jimenez-Andrade et al., 2006; Peters et al., 2007; Barrière et al., 2012; Doyle et al., 2012). Overproduction of peroxynitrite contributes to increasing neuro-excitatory and pro-inflammatory cytokines (TNF-alpha and IL-1beta) and to decreasing anti-inflammatory cytokine (IL-10 and IL-4) production (Ledeboer et al., 2007; Doyle et al., 2012).

*In vitro* studies highlighted that taxanes induced neuropathic symptoms by inhibiting anterograde fast axonal transport (conventional kinesin-dependent) (LaPointe et al., 2013) in the peripheral endings of sensory neurons and altering neurotransmitter release (Carozzi et al., 2010; Gracias et al., 2011; Gilardini et al., 2012). Taxane treatment revealed reversible enlargement of the nucleoli of sensory neurons after a single-dose of paclitaxel in rat (Jamieson et al., 2003). Furthermore, Jimenez-Andrade et al. (2006) reported that a cumulative dose of 36 mg/kg of paclitaxel in rat significantly increased the number of ATF-3 (a cell injury marker) neurons in trigeminal ganglia and DRG. Mitochondrial alterations caused by the production of reactive oxygen species in peripheral nerves were also shown to be closely related to neuropathic effects (Barrière et al., 2012; Xiao and Bennett, 2012). Other studies also recently reported an increase of the toll-like receptor TLR4 and its immediate downstream signaling molecules, myeloid differentiation primary response gene 88 (MyD88), and TRIF, in DRG after paclitaxel treatment in favor of a pro-inflammatory mechanism (Li et al., 2014, 2015). The activation of TLR4 was associated with the sensitization of transient receptor potential vanilloid subtype 1 (Hara et al., 2013; Li et al., 2015) known to be involved in nociception (**Figure 1**).

### Symptoms and Long-Term Effects of CIPN Associated to Taxanes

Few studies have reported the increased incidence of acute and chronic toxicities with taxanes that could potentially lead to dose reductions and treatment withdrawal (Tanabe et al., 2013; Ho and Mackey, 2014). It is difficult to know whether paclitaxel or docetaxel is the most neurotoxic as the scientific literature on the topic is unclear and contradictory (Jones et al., 2005; Shimozuma et al., 2012). Neurophysiological examinations of patients with CIPN revealed a decrease in sensory nerve conduction velocity and compound action potential amplitude (Chaudhry et al., 1994; Dougherty et al., 2004). This CIPN is a typical distal sensory neuropathy with a stocking-and-glove distribution over the hands and feet. The patients can report paresthesia, dysesthesia, numbness and altered proprioception. Motor weakness of hands and feet are less frequent, such as vegetative disturbances (De Iuliis et al., 2015). Taxane-associated CIPN is considered to be a good predictor of neuropathic pain after paclitaxel treatment, as 27% of those patients with CIPN experienced neuropathic pain (Reyes-Gibby et al., 2009). These complications are often the main reason for treatment cessation (Tanabe et al., 2013). However, symptoms may aggravate after the end of the chemotherapy (De Iuliis et al., 2015) (**Table 1**).

Among patients treated with adjuvant paclitaxel chemotherapy, between 80 to 97% experienced symptoms of neuropathy with a time range to CIPN onset of 1–101 weeks (Chaudhry et al., 1994; Hershman et al., 2011; Tanabe et al., 2013). These symptoms remained during a median follow-up time of 57 months for 212 neuropathic patients (minimum: 5.3, maximum: 95.5) (Tanabe et al., 2013). In a Multicenter Italian Trial in Ovarian cancer (MITO-4), 22 out of 60 neuropathic patients (37%) treated with carboplatin and paclitaxel reported complete recovery in the first 2 months after the end of the chemotherapy (Pignata et al., 2006). Nevertheless, 15 patients (25%) recovered between 2 and 6 months, and nine patients (15%) after 6 months and more. Regarding a 46-patient cohort (ACCRU pilot trial) treated with paclitaxel alone, similar results were reported based on the sensory neuropathy scores of the QLQ-CIPN20 of the EORTC (Shinde et al., 2016). In a study conducted in the Netherlands, most of the patients complained about neurotoxicity in the upper and lower extremities 6 months after cessation of chemotherapy with oxaliplatin, paclitaxel, or docetaxel (78.8 and 89.7%, respectively) (Beijers A. et al., 2014). These neuropathies primarily included numbness and tingling in hands, feet, suffering from cold feet, and trouble distinguishing objects with the hands.

A significant correlation was found between scores on emotional well-being and neuropathy symptoms with the FACT/GOG-Ntx (Beijers A. et al., 2014). Another study demonstrated that persistent limb pain linked to docetaxel treatment was responsible for the deterioration of psychological function (Ventzel et al., 2016). More recently, breast cancer survivors with CIPN developed more severe insomnia, anxiety, and depression than those without neuropathy (Bao et al., 2016). Thornton et al. (2008) demonstrated that in the years following chemotherapy, the taxane group had significantly worse emotional distress and mental HRQOL throughout adjuvant treatment. These outcomes were also associated with rates of probable clinical depression during the first year. The taxane cohort had a significantly slower psychological recovery and required 2 years on average for emotional recovery compared with 6–12 months for patients in the no taxane comparison group (Thornton et al., 2008). Another longitudinal study monitoring HRQOL parameters in a 6-year study found similar results with a return to baseline within 2 years and no change at 6 years (Hall et al., 2014). Finally, 15% of breast cancer survivors reported CIPN with a significant impact on HRQOL scales, from 1 to 3 years after a single docetaxel containing regimen (Eckhoff et al., 2015). Interestingly, the relative tolerability of regimens according to HRQOL assessment was equivalent between the two single-agent docetaxel and paclitaxel treatments. However, the mean neurotoxicity related subscale FACT/GOG-Ntx from baseline to 1 year following the end of treatment were significantly more severe for the paclitaxel-treated group compared to the docetaxel-treated group (Shimozuma et al., 2012).

### CIPN Risk Factors Associated to Taxanes

Although the severity of taxane-mediated CIPN differs as a function of several demographic variables, it is very difficult to predict which patients will develop this CIPN (Schneider et al., 2015). Demographic variables such as health status, obesity, and age are known to predispose to neuropathy (Hershman et al., 2011; Schneider et al., 2012) and influence CIPN duration (Tanabe et al., 2013). Indeed, patients with pre-existing neuropathy (related to diabetes, alcohol or even idiopathic) developed severe neuropathy after receiving taxane-based chemotherapy (Rowinsky et al., 1993; Chaudhry et al., 2003). Cumulative dose (135–1400 mg/m^2^) may be a risk of CIPN, but this parameter remains a subject of debate in the literature (van Gerven et al., 1994; Tanabe et al., 2013). Tanabe et al. (2013) did not find any relation between neuropathy grade and diabetes, or with radiotherapy, dose intensity and cumulative dose. CIPN severity may differ depending on chemotherapy type and protocol (Schneider et al., 2012; Shimozuma et al., 2012; Tanabe et al., 2013). Indeed, the median time to neuropathy onset seemed to be inversely correlated with the tighter administration schedule, 35 and 21 days for weekly and tri-weekly administration, respectively (Tanabe et al., 2013). Consequently, the pharmacokinetics of taxanes impact their neurotoxicity and a pharmacokinetic-based dosing algorithm has been proposed to reduce paclitaxel-related neurotoxicity (Kraff et al., 2015).

The heterogeneity of taxane-induced neuropathy is probably not only associated with demographic variables alone, suggesting contributions of genetic variability. Investigations suggest a possible genetic predisposition to the occurrence of taxane-induced peripheral neuropathy (Frederiks et al., 2015). Recent studies examined the effect of SNP in the congenital peripheral neuropathy gene FGD4 as a genetic susceptibility to neuropathic disorders (Baldwin et al., 2012). This SNP, and markers in additional genes [including EPHA5 (rs7349683) and FZD3 (rs10771973)], were associated with the onset or severity of paclitaxel-induced peripheral neuropathy. Additional experiments examined the pharmacokinetic profiles of taxanes to establish whether exposure to them is correlated with the degree of neurotoxicity (Gréen et al., 2009; de Graan et al., 2013). The pharmacokinetic profiles of docetaxel in 50 patients with 59 different SNPs (including tag-SNSps and PXR/NR1I2, CAR/NR1I3, RXRα/NR2B1, HNF4α/NR2A1 genes) were characterized by marked interindividual variability, with approximately four- to six-fold variations observed at maximal concentration, AUC and plasma clearance (Chew et al., 2013). Another clinical study found that docetaxel clearance was also modulated in patients carrying the CYP3A^∗^1B allele or GSTP1^∗^A/^∗^B and 3435TT genotypes (Tran et al., 2006). Further works demonstrated that, among 13 relevant polymorphisms in genes encoding paclitaxel metabolizing enzymes, CYP2C8 haplotype C and CYP3A5^∗^3 were associated with neuroprotection and the clearance of paclitaxel, and conversely with an increased risk of neuropathy (Gréen et al., 2009; Leskelä et al., 2011; Hertz et al., 2013). However a recent study underlined a sexual dimorphism with CYP3A4: women carrying the CYP3A4^∗^22 allele had increased risk of developing severe neurotoxicity during paclitaxel treatment (de Graan et al., 2013). Other studies demonstrated an involvement of ABCB1 gene polymorphisms encoding for *P*-glycoprotein, a primary protein involved in taxane elimination and distribution, with neuropathy in metastatic breast cancer patients treated with paclitaxel or docetaxel monotherapy. Patients treated with docetaxel carrying another ABCB1 2677GG genotype had a significantly longer time to neuropathy (Sissung et al., 2008). Indeed patients heterozygous for G/A in position 2677 in ABCB1 had significantly higher toxic clearance than most other ABCB1 variants (Gréen et al., 2009). Another study underlined that patients carrying two reference alleles for ABCB1 3435CT polymorphism tended toward a reduced risk of developing CIPN compared to patients carrying only one allele (Sissung et al., 2006) (**Table 2**).

## Vinca Alkaloids

Vinca alkaloids are plant-derived microtubule assembly inhibitors originally derived from the periwinkle *Catharanthus roseus* (Liu et al., 2014). Vinca alkaloids are now produced synthetically and are used in particular in the treatment of acute lymphoblastic leukemia, Hodgkin’s disease, non-Hodgkin lymphoma, and many cancers (rhabdomyosarcoma, osteosarcoma, uterus, breast, lung, etc.). They may be used in mono-chemotherapy and poly-chemotherapy treatments. The vinca alkaloid family includes vinblastine, vinorelbine, vindesine, and vincristine (Liu et al., 2014).

### Pathophysiological Mechanisms of CIPN Associated to Vinca Alkaloids

Vinca alkaloids block microtubule polymerization, by binding to free tubulin dimers (β-α-tubulin heterodimers interface) close to the GTP-binding sites (vinca domain) (Jordan and Kamath, 2007), inducing an increase of microtubule depolymerization and inhibiting the hydrolysis of GTP, which stops the mitotic cycle and initiates cell apoptosis (Jordan and Wilson, 2004; Liu et al., 2014).

Due to their cytotoxic action, vinca alkaloids induce many adverse effects of which neurotoxicity remains the most frequent. This neurotoxicity affects the neuronal cytoskeleton which causes axonal degeneration and the impairment of axonal transport. For unknown reasons, the sensory fibers are reached earlier, more frequently and more severely than the motor fibers. The complete mechanism of vinca alkaloid-induced peripheral neuropathy involves several actors:

- Endogenous opioids, which play a critical role in nociception, such as endomorphin-2, are decreased in the spinal cord and DRG of animals treated with vincristine, without mu-opioid receptor expression change. This contributes to the development of neuropathic pain symptoms, leading to hypersensitivity of C-fiber nociceptors and abnormal activity of the wide dynamic range neurons (Yang et al., 2014). Oxidative stress, generated after the impairment of mitochondrial function and the overproduction of reactive oxygen species following vinca alkaloid-based treatment, influences the activity of serine protease, which inactivates endomorphins in the spinal cord, thus suggesting that oxidative stress is a key mechanism of this CIPN (Wang et al., 2008).
- Spinal synaptic plasticity involved in the maintenance of neuropathic symptoms is also related to this CIPN. C-Fos (a marker of neuronal activation) and Piccolo (maintenance of synaptic plasticity) were increased in the neurons of the spinal cord in CIPN animal models, suggesting increased neuronal activity and a structural reorganization of pre-synaptic elements (Ibi et al., 2010; Thibault et al., 2013).
- Central glia (astrocytes and microglia) plays a critical role in neuropathic symptoms and its inhibition remains a potential strategy for alleviating these symptoms (Watkins and Maier, 2005). In an animal model of vincristine-induced peripheral neuropathy, astrocyte activation participates in neuropathic symptoms through the up-regulation of interleukin-1β and NMDA sensitization (phosphorylation induced by interleukin-1β) (Ji et al., 2013).
- Serotonin transporter null mice elicit reversed neuropathic pain behavior in animal models of vincristine-associated CIPN. Considering that serotonin has an influence on pain transmission, also in CIPN animal models (Suzuki et al., 2004), this impact on neuropathic symptoms may be attributed to a lack of spinal serotonin. Furthermore, tropisetron (a selective antagonist of serotonin receptor of type 3) is able to ameliorate vincristine-induced peripheral neuropathy in rat (Barzegar-Fallah et al., 2014).
- Vinca alkaloids can alter calcium homeostasis through the dysregulation and structural modification of mitochondria, decreasing the amount and rate of calcium uptake and efflux (Tari et al., 1986). These changes induce increased neuronal excitability and impaired glial function. Moreover, neuropathic symptoms produced by vinca alkaloids are alleviated by drugs that decrease the extracellular and intracellular availability of calcium (Siau and Bennett, 2006).
- Jaggi and Singh (2012) demonstrated a link between MAPK and vincristine-induced peripheral neuropathy. In this study, the antineuropathic effects of farnesyl thiosalicylic acid (Ras inhibitor) and GW5074 (c-Raf1 kinase inhibitor) in an animal model of vincristine-induced peripheral neuropathy was demonstrated (Jaggi and Singh, 2012). This result suggests that Ras and c-Raf-1 are potential targets for preventing CIPN (**Figure 1**).

### Symptoms and Long-Term Effects of CIPN Associated to Vinca Alkaloids

Vinca alkaloids induce glove-and-stocking distribution peripheral neuropathy in 35% to 45% of patients (Postma et al., 1993; Verstappen et al., 2005). Sensory neuropathy typically develops first in the hands and feet, leading to functional disability with fine motor tasks and walking, including numbness and tingling (Postma et al., 1993; Verstappen et al., 2005; Boyette-Davis et al., 2013). These symptoms often develop after several weeks of treatment but can occur after the first dose. The coasting effect was also prominent in vinca alkaloid-induced peripheral neuropathy, with 30% of patients subject to worsening symptoms after stopping treatment (Haim et al., 1994). In addition to sensory symptoms, motor and autonomic neuropathies were also prominent. Neuropathic patients experienced muscle cramps and distal muscle weakness (Haim et al., 1994). Autonomic symptoms include heart rate variability reduction (Hirvonen et al., 1989), postural hypotension, bladder and bowel disturbance, ocular palsies and vocal cord paralysis (Hancock and Naysmith, 1975; Quasthoff and Hartung, 2002). Vinca alkaloid treatment was also associated with acute motor neuropathy, similar to the Guillain–Barré syndrome (González Pérez et al., 2007). Vinca alkaloids are frequently used in pediatric hematological malignancies. Lavoie Smith et al. (2015) made an interesting assessment of the vincristine-induced peripheral neuropathy in children treated for acute lymphocytic leukemia. In these children, 78% developed a sensory-motor CIPN and 44% reported pain. Overall severity was low, but a subgroup of children developed severe forms of CIPN. The main identified symptoms were decrease of reflexes, vibration sensibility, and strength (Lavoie Smith et al., 2015). In another study on children and adolescents treated for non-CNS solid and hematological malignancies, up to 85% of vincristine-treated children suffered of CIPN during treatment and 40% at 6 months post-treatment. Higher symptoms and deficits were found for patients treated for lymphoma or solid tumors compared to acute lymphocytic leukemia (Gilchrist et al., 2017) (**Table 1**).

Neuropathy has been described to be reversible and mainly resolved within 2 months (Haim et al., 1994), although some patients report lasting dysfunction with sensory symptoms persisting longer than motor symptoms (Postma et al., 1993; Boyette-Davis et al., 2013). Nevertheless, long-term follow-up of patients who received vinca alkaloid treatment revealed that 32% had sensory symptoms which persisted from 34 to 48 months after treatment (Postma et al., 1993; Boyette-Davis et al., 2013) and 14% had disabling sensory neuropathy 9 years after treatment (Moser et al., 2005). Another study by Oerlemans et al. (2014) demonstrated that patients with diffuse large B-cell lymphoma present neuropathic symptoms for up to 5 years (tingling hands/feet are described in 30% of patients). In comparison, 30–34% of children with acute lymphoblastic leukemia had neuropathic symptoms from 3 to 7 years following vinca alkaloid chemotherapy (Ramchandren et al., 2009; Jain et al., 2014).

Only two studies assessed the impact of this CIPN on the HRQOL of patients. Both studies used either the SF-36 or the QLQ-C30 from the EORTC questionnaires to evaluate HRQOL. In the study by Kim et al. (2010) patients with sensory neuropathy following vinca alkaloid-based treatment (18 weeks) reported a lower HRQOL than those without neuropathy. The SF-36 questionnaire demonstrated that neuropathic patients had impaired physical functions and lower vitality than non-neuropathic patients. No change was observed for the other items (bodily pain, general health, social function and general mental health). In their study Liew et al. (2013) observed that global health was similar to normative data 28 months after completing vinca alkaloid-based chemotherapy, but leukemia survivors had lower cognitive and social functions and reported more financial difficulty. Fatigue and pain affected 83 and 53% of patients, respectively, and both showed significant inverse correlation with overall health and all functional scales. Nevertheless, although therapy-related symptoms were persistent, long-term survivors had a global HRQOL similar to that of the general population (Liew et al., 2013). Overall, these studies indicated that persistent neuropathy has a considerable impact on patients’ lives.

### CIPN Risk Factors Associated to Vinca Alkaloids

Antifungal treatment with azole-based agents may exacerbate neuropathy via the inhibition of the cytochrome CYP3A involved in vinca alkaloid metabolism (Moriyama et al., 2012). Thus the relationship between genetic factors related to CYP3A, and CIPN has been investigated by several studies. Egbelakin et al. (2011) demonstrated that CYP3A5 expression was related to CIPN in acute lymphoblastic leukemia in children. Indeed, a child with CYP3A5 genotype develops less peripheral neuropathy compared to CYP3A5 non-expressers. Another study demonstrated that acute CIPN was related to the presence of SNPs of genes involved in the cell cycle and cell proliferation, such as GLI1 (rs2228224 and rs2242578), and to the up-regulation of other genes participating in the cell cycle and cell proliferation such as aurora kinase A and the marker of proliferation Ki-67 (Broyl et al., 2010). Moreover, the chronicity of neuropathic symptoms was associated with SNPs in genes involved in absorption, distribution, metabolism, and excretion (Broyl et al., 2010). In addition, 17p11.2-12 duplication (associated with CMT1A) has been demonstrated to be a predictor of severe neurotoxicity in patients (Graf et al., 1996), and more widely, several studies have demonstrated a higher risk of inducing severe acute neurotoxicity in patients with CMT1A (Naumann et al., 2001; Orejana-García et al., 2003; Nishikawa et al., 2008). Finally, sensory neuropathy is less rare in patients expressing a variant of the gene Centrosomal Protein 72 (Diouf et al., 2015).

The occurrence of neuropathy was also strongly dose-dependent, with development at a dose of 2–6 mg/m^2^ (Postma et al., 1993; Haim et al., 1994; Verstappen et al., 2005). It is noteworthy that neurotoxicity can occur with a single dose in patients receiving a 4 mg dose and demonstrating worse neurotoxicity than those receiving a 2 mg dose (Verstappen et al., 2005). Thus, total dose levels have been capped at 2 mg/m^2^ regardless of body surface area (Haim et al., 1994). For vincristine treated children, older patients are at higher risk of sensory and motor CIPN. Sex is more debated, but female would be at higher risk for CIPN (Lavoie Smith et al., 2015; Gilchrist et al., 2017) (**Table 2**).

## Bortezomib and Thalidomide

Bortezomib, a dipeptidyl boronic acid, is the first of a new class of proteasome inhibitors approved in 2004 by both US and European authorities for the treatment of multiple myeloma and in 2006 for the treatment of mantle cell non-Hodgkin’s lymphoma (Argyriou et al., 2014). Bortezomib is the cornerstone treatment for multiple myeloma, and is commonly used to treat newly diagnosed as well as relapsed/refractory multiple myeloma, either as single agent or combined with other therapies, leading to a major improvement in disease management and increasing the lifespan of patients.

Thalidomide is a glutamic acid derivative and an oral immunomodulatory and antiangiogenic agent. It was the first drug designed to treat nausea in pregnant woman in the 1960s. Widely known for its teratogenic effects, thalidomide was approved by the US Food and Drug Administration for the treatment of multiple myeloma (Richardson et al., 2002).

### Pathophysiological Mechanisms of CIPN Associated to Bortezomib and Thalidomide

Both thalidomide and bortezomib exert pleiotropic actions, which complicates understanding of their neurotoxic effects (Morawska et al., 2015). Little is known at present about the mechanism underlying this neurotoxicity.

The pathogenetic hallmark of bortezomib-induced peripheral neuropathy consists of morphological alterations in the spinal cord, DRG and peripheral nerves, with specific functional alterations in Aδ and C sensory nerve fibers (Carozzi et al., 2013; Staff et al., 2013). In addition, proteasome inhibition increased a-tubulin polymerization, mitochondrial and endoplasmic reticulum damage, and dysregulation of neurotrophins through the inhibition of NF-kB (nuclear factor kappa B) activation may also significantly contribute to this CIPN genesis (Landowski et al., 2005; Staff et al., 2013). However, these findings do not explain why preferentially thin and unmyelinated nerve fibers are affected. It was recently suggested that bortezomib-induced peripheral neuropathy occurs via a proteasome-independent mechanism (Arastu-Kapur et al., 2011), possibly involving mitochondrial dysfunction (Zheng et al., 2012). By contrast with immunomodulatory drugs, a more specific neurotoxic action of bortezomib occurs through the transient release of intracellular calcium stores, leading to mitochondrial calcium influx and caspase-induced apoptosis (Landowski et al., 2005). Disruption of intracellular calcium homeostasis in nerves can promote depolarization and spontaneous discharge, causing pain and other abnormal sensations. Finally, a higher ratio of polymerized versus soluble tubulin was found in neural cells after treatment with proteasome inhibitors, suggesting a mechanism by which this neurotoxic anticancer drug could interfere with microtubular stability (Poruchynsky et al., 2008).

Thalidomide has several actions, including a role in modifying integrin receptors, altering TNFα and inhibiting angiogenesis. The mechanism of action of thalidomide on malignant cells is poorly understood but may involve both immunomodulation and antiangiogenic effects, resulting in partially irreversible damage to distal axons, DRG neurons and central projections of primary afferent neurons (Giannini et al., 2003). Because thalidomide has antiangiogenic activities, it was initially proposed that one of the mechanisms of thalidomide-induced peripheral neuropathy was capillary damage and secondary anoxemia in nerve fibers. Additionally, it was suggested that thalidomide reduces neural cell survival by downregulation of TNFα, triggering inhibition of NF-kB and subsequent acceleration of neuronal cell death (Fernyhough et al., 2005). NF-kB inhibition is one of the main effects of bortezomib and could provide a common link between the neurotoxicity of thalidomide and proteasome inhibition. A crucial event in thalidomide-induced peripheral neuropathy may be the suppression of NF-kB, a factor linked to p65 (activated by TNFα) and p75 (activated by pro-neurotrophins) receptors (Li et al., 2009). These receptors may induce both apoptosis and cell growth, depending on the circumstances (Ibáñez and Simi, 2012) (**Figure 1**).

### Symptoms and Long-Term Effects of CIPN Associated to Bortezomib and Thalidomide

Although bortezomib and thalidomide represent a major advance in the treatment of multiple myeloma, they are also unfortunately accompanied by an increase of challenging treatment-related adverse events; in particular they frequently induce dose-limiting peripheral neuropathy. Bortezomib-induced peripheral neuropathy is considered to be one of the most severe, unpredictable and potentially permanent non-hematological side-effects of chemotherapy against multiple myeloma. Thus it also has a detrimental effect on the HRQOL of survivors (Argyriou et al., 2014) and compromises optimal treatment for patients with multiple myeloma. Although rare, autonomic peripheral neuropathy can be life threatening, leading to serious medical conditions such as irregular heartbeat, hypotension, and shortness of breath. The incidence of this CIPN (any grade) in large clinical studies ranges from 31 to 64% (Richardson et al., 2003, 2005; Velasco et al., 2010) for bortezomib and from 10 to 55% for thalidomide (Jongen et al., 2015). The data collected showed a higher percentage of patients developing CIPN following thalidomide at doses of 200 mg/day or higher in comparison to lower thalidomide doses (Glasmacher et al., 2006).

Bortezomib-induced peripheral neuropathy is typically a predominantly sensory axonopathy associated with burning dysesthesia, coldness, numbness, hyperesthesia, and/or tingling in a distal stocking-and-glove distribution over the hands and feet. Neuropathic pain is a prominent feature of this CIPN, occurring in 25–80% of cases (Rampen et al., 2013), characterized by shooting pain and severe cramps, due to dysfunction of all three major sensory nerve fibers (Aβ, Aδ, and C), as demonstrated in both clinical and animal models (Cata et al., 2007). Signs and symptoms of autonomic dysfunction may occur, since these are also served by unmyelinated nerve fibers. Autonomic dysfunctions are present in 12–50% of patients, with constipation and orthostatic hypotension being the most frequent symptoms (Velasco et al., 2010). Other autonomic disturbances are frequently observed and lead to adverse gastrointestinal events (Richardson et al., 2010). Motor fibers are rarely affected (Mateos, 2012). Bortezomib-induced peripheral neuropathy is an early complication when it occurs shortly after the introduction of treatment. CIPN generally occurs during the first 5 cycles of bortezomib treatment and is related to cumulative dose and reaching a plateau at cycle 5 (Richardson et al., 2009) (**Table 1**).

As with bortezomib, thalidomide-induced peripheral neuropathy causes often painful distal sensory axonal peripheral neuropathy in over half of patients if treated over a sufficiently long period of time (Cavaletti et al., 2004). Thalidomide-induced peripheral neuropathy affects large and small fibers, associated with tingling or painful paresthesia, and numbness in the lower limbs (Plasmati et al., 2007). Its onset is usually slower than for bortezomib. Mild motor impairment also appears to be present (Chaudhry et al., 2008), but is only significant in severe cases (Giannini et al., 2003). Autonomic manifestations, including gastrointestinal (constipation, anorexia, and nausea), and cardiovascular (hypotension and bradycardia) effects are commonly observed (Morawska et al., 2015). A dual role for thalidomide has been highlighted by clinical observations that suggest that thalidomide may be neuroprotective in patients receiving a combination with bortezomib, while being neurotoxic when given as a single agent (Badros et al., 2007). This might be explained by its anti-inflammatory effect in preventing excess neurotoxicity (**Table 1**).

Unfortunately, these CIPNs are not always reversible. Although reversal of bortezomib-induced peripheral neuropathy after treatment cessation is frequent, recovery in some patients may take months, up to 2 years, and some will never fully recover neurological function (Cavaletti and Jakubowiak, 2010). This CIPN has a significant impact on HRQOL, including the physical, social, and psychological effects of unrelieved pain (Tariman et al., 2008). At present, extensive reports on the long-term evolution of this CIPNs are not available. The long-term evolution of thalidomide-induced peripheral neuropathy has not yet been studied extensively, although it is suggested that this CIPN may improve after thalidomide dose-reduction or discontinuation (Argyriou et al., 2012). However, some patients may be subject to permanent damage (Mohty et al., 2010).

Maximizing the benefits of treatment while preserving HRQOL therefore requires a careful balance between achieving optimum activity and minimizing toxicity, in order to further enhance efficacy. Monitoring for signs and symptoms of peripheral neuropathy during bortezomib or thalidomide therapy, such as the Indication for Common Toxicity Criteria Grading of Peripheral Neuropathy Questionnaire, should ensure early recognition, allowing for prompt dose reduction and discontinuation which are the mainstays of preventing and managing CIPN, and increasing the probability of recovery of patients undergoing cancer treatment (Beijers et al., 2016).

### CIPN Risk Factors Associated to Bortezomib and Thalidomide

Risk factors for CIPN in multiple myeloma patients include advanced age, prior neuropathy and drug combinations, but not genetic factors (García-Sanz et al., 2016). Nevertheless, in larger studies, baseline neuropathy was the only consistent risk factor for bortezomib-induced peripheral neuropathy. Age, diabetes, International Staging System stage, obesity, and creatinine clearance did not affect the overall rate of this CIPN (Dimopoulos et al., 2011; Tacchetti et al., 2014). Like almost any neurotoxic antineoplastic drug, the cumulative bortezomib dose is the most significant risk factor of CIPN development. The study by Tacchetti et al. (2014) demonstrated that the rate of grade 2 CIPN in the VTD arm was three times higher than in the thalidomide dexamethasone arm of the study. Of all the treatment phases, induction therapy was associated with the highest risk of CIPN, while the lowest risk was related to consolidation therapy (Tacchetti et al., 2014). Furthermore, lowering the dose of bortezomib to 1 mg/m^2^ was associated with a reduced risk of developing severe neurological toxicity after four cycles of VTD (Moreau et al., 2011a). Since 2012, The Food and Drug Administration and the European Medicine Agency have validated the subcutaneous injection of bortezomib instead of the intravenous injection in order to limit the adverse effects and CIPN (Moreau et al., 2011b; Minarik et al., 2015). However, a recent study show that the prevalence and severity of bortezomib-induced peripheral neuropathy were not different between intravenous and subcutaneous ways (Minarik et al., 2015) (**Table 2**).

## Conclusion

As presented in this review, CIPN represents a very problematic adverse event of certain anticancer chemotherapies. First, these CIPNs are frequent in cancer patients treated with neurotoxic anticancer drugs with an overall incidence of approximately 38% (possibly as many as 90% of patients treated with oxaliplatin). Finally, the long-term reversibility of these CIPNs remains questionable, notably in the case of platinum-based anticancer drugs and taxanes, for which CIPN may last several years after the end of anticancer chemotherapies. These CIPN are also very problematic for young patients (children, adolescents, and young adults), which may interfere with their own development and social life. As we have seen, these long-term effects are associated with comorbidities such as depression, insomnia and a decrease of HRQOL in cancer patients and survivors. However, it is noteworthy that these long-term effects remain poorly studied, and only limited data are available such as in the case of bortezomib and thalidomide-induced peripheral neuropathy, despite the shorter life expectancy of patients.

Some risk factors of CIPN have been identified for each anticancer drug. The most applicable ones are the control of cumulative doses, preexisting neuropathic disorders and age of patients. But these preventive measures have a limited effect in clinical practice because patients still suffer of CIPN. No preventive or curative pharmacological strategy has yet been acknowledged. This can be explained by the fact that the drugs chosen to treat or prevent CIPNs are the same as those used to treat common neuropathic pain conditions, such as nerve injury, post-herpetic neuralgia, polyneuropathy, and painful diabetic peripheral neuropathy. The main preclinical and clinical studies have been performed although CIPN differs from other forms of neuropathy, particularly in terms of pathophysiology and symptomatology. CIPNs are frequently associated with sensory symptoms (numbness, tingling) without severe neuropathic pain symptoms (shooting/burning pain), point that we recently debated in the literature (Kerckhove et al., 2017).

For many patients, these CIPNs are not vital adverse effects but impact greatly their quality of life, and for cancer survivors, these CIPN are reminders of the cancer disease and its treatments. Oncologists decrease or stop neurotoxic anticancer drugs, thus limiting the severity of these neurological symptoms “and that’s all.” However, given the major improvement of the therapeutic management of many cancers and the increasing number of cancer survivors, it is now urgent to discover new and effective strategies to prevent and/or treat these CIPNs and their long-term effects.

## Author Contributions

NK, AC, SC, CC, DP, and DB contributed to the design of the manuscript and the acquisition of data from the literature. All the authors participated in drafting and revising the manuscript they all approved the final version of the manuscript for submission.

## Conflict of Interest Statement

The authors declare that the research was conducted in the absence of any commercial or financial relationships that could be construed as a potential conflict of interest. The reviewer ES-R and handling Editor declared their shared affiliation, and the handling Editor states that the process nevertheless met the standards of a fair and objective review.

## Abbreviations

ABCB1: ATP binding cassette B1
AUC: area under the curve
BCRP: breast cancer resistance protein
CIPN: chemotherapy-induced peripheral neuropathy
CMT1A: Charcot-Marie-Tooth disease type 1A
DRG: dorsal root ganglia
EORTC: European Organisation for Research and Treatment of Cancer
FACT/GOG-Ntx: functional assessment of chronic illness therapy/gynecologic oncology group-neurotoxicity
GFAP: glial fibrillary acidic protein
GST: glutathione-*S*-transferase
GTP: guanosine triphosphate
HCN: hyperpolarization-activated cyclic nucleotide-gated
HRQOL: health-related quality of life
KCN: potassium channel
MAPK: mitogen-activated protein kinase
MyD88: myeloid differentiation primary response gene 88
NaV: voltage-gated sodium channel
NF-kB: nuclear factor kappa B
OCT2: organic cation transporter 2
PNS: peripheral nervous system
QLQ-C30: quality of life core questionnaire
QLQ-CIPN20: quality of life questionnaire to assess chemotherapy-induced peripheral neuropathy
SF-36: 36-item short form health survey
SNP: single nucleotide polymorphism
TLR: toll-like receptor
TRAAK: TWIK-related arachidonic acid activated K^+^ channel
TREK1: TWIK-related K^+^ channel 1
TRIF: toll/interleukin 1 receptor domain–containing adapter-inducing interferon-b
TRPM8: transient receptor potential melastatin 8
TNFα: tumor necrosis factor alpha
TWIK: tandem of P domains in a weak inward-rectifier K^+^ channel
VTD: Velcade Thalidomide Dexamethasone
